# Maternally transmitted nonsyndromic hearing impairment may be associated with mitochondrial tRNA^Ala^ 5601C>T and tRNA^Leu(CUN)^ 12311T>C mutations

**DOI:** 10.1002/jcla.24298

**Published:** 2022-02-26

**Authors:** Xuejiao Yu, Sheng Li, Yu Ding

**Affiliations:** ^1^ Department of Clinical Laboratory Quzhou People's Hospital the Quzhou Affiliated Hospital of Wenzhou Medical University Quzhou China; ^2^ Department of Otolaryngology Quzhou People's Hospital the Quzhou Affiliated Hospital of Wenzhou Medical University Quzhou China; ^3^ Central Laboratory Hangzhou First People’s Hospital Zhejiang University School of Medicine Hangzhou China

**Keywords:** m.12311T>C, m.5601C>T, mitochondrial dysfunctions, mt‐tRNA mutations, NSHL

## Abstract

**Background:**

Sequence alternations in mitochondrial genomes, especially in genes encoding mitochondrial tRNA (mt‐tRNA), were the important contributors to nonsyndromic hearing loss (NSHL); however, the molecular mechanisms remained largely undetermined.

**Methods:**

A maternally transmitted Chinese pedigree with NSHL underwent clinical, genetic, and biochemical assessment. PCR and direct sequence analyses were performed to detect mitochondrial DNA (mtDNA), *GJB2*, and *SLC26A4* gene mutations from matrilineal relatives of this family. Mitochondrial functions including mitochondrial membrane potential (MMP), ATP, and ROS were evaluated in polymononuclear leukocytes (PMNs) derived from three deaf patients and three controls from this pedigree.

**Results:**

Four of nine matrilineal relatives developed hearing loss at the variable age of onset. Two putative pathogenic mutations, m.5601C>T in tRNA^Ala^ and m.12311T>C in tRNA^Leu(CUN)^, were identified via PCR‐Sanger sequencing, as well as 34 variants that belonged to mtDNA haplogroup G2b2. Intriguingly, m.5601C>T mutation resided at very conserved nucleotide in the TψC loop of tRNA^Ala^ (position 59), while the T‐to‐C substitution at position 12311 located at position 48 in the variable stem of tRNA^Leu(CUN)^ and was believed to alter the aminoacylation and the steady‐state level of tRNA. Biochemical analysis revealed the impairment of mitochondrial functions including the significant reductions of ATP and MMP, whereas markedly increased ROS levels were found in PMNs derived from NSHL patients with m.5601C>T and m.12311T>C mutations. However, we did not detect any mutations in *GJB2* and *SLC26A4* genes.

**Conclusion:**

Our data indicated that mt‐tRNA^Ala^ m.5601C>T and tRNA^Leu(CUN)^ 12311T>C mutations were associated with NSHL.

## Graphical abstract

In this case–control study for genetic screening of deafness‐associated mitochondrial tRNA mutations/variants, we ascertained one maternally inherited Han Chinese family harboring mitochondrial tRNA^Ala^ C5601T and tRNA^Leu(CUN)^ T12311C mutations. We further screened the whole mitochondrial genomes of the matrilineal relatives from this pedigree. In addition, the phylogenetic conservation analysis and mtDNA haplogroup analysis were performed. We further isolated the polymononuclear leukocytes and performed the mitochondrial functional analysis including ATP, mitochondrial membrane potential, and ROS in patients carrying m.C5601T and m.T12311C mutations. We found that m.C5601T and m.T12311C mutations may cause mitochondrial dysfunction, which was involved in the pathogenesis of maternally inherited hearing loss.

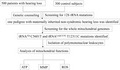

## 1 INTRODUCTION

Deafness was a common communication disorder affecting ~360 and 27 million individuals all over the world and in China, respectively.^1^ Genetic impact had been found >50% patients with hearing loss. To date, around 124 genes, as well as 1,000 mutations, had been identified to be related to NSHL (https://hereditaryhearingloss.org/).^2^, ^3^ Of these nuclear genes, mutations in *GJB2*,^4^ *GJB3*,^5^ *GJB6*,^6^ *NCOA3*,^7^ *SLC26A4*,^8^ and *POU4F3* ^9^ were the most important causes for hearing impairment. In addition to the nuclear gene mutations, mitochondrion was very important organelle whose primary role was to generate ATP via oxidative phosphorylation (OXPHOS). Moreover, mitochondria had their own genetic codes, named mtDNA, which was 16,569 bp in length.^10^ Mutations in mtDNA played important roles in the progression of NSHL.^11^, ^12^ In particular, the well‐known m.1555A>G and m.1494C>T substitutions in the A site of 12S rRNA gene had been found in patients with both aminoglycoside‐induced and NSHL.^13^, ^14^ Additionally, increasing evidence suggested that mt‐tRNA genes mutations were associated with deafness.^15^, ^16^, ^17^ In fact, tRNA^Leu(UUR)^ 3243A>G was the most common pathogenic mutation for syndromic hearing loss.^18^ Furthermore, tRNA^Ser(UCN)^ 7445A>G, 7505T>C, 7510T>C, and 7511T>C,^19^ and tRNA^His^ 12201T>C mutations^20^ were associated with NSHL in families worldwide. Mutations in mt‐tRNA may decrease the steady‐state level of mt‐tRNA and impair mitochondrial protein synthesis.^21^ Possibly molecular mechanisms underlying these mt‐tRNA mutations may be the abnormal mt‐tRNAs processing, affecting epigenetic modifications or influencing the interactions between mt‐tRNA and other transcriptional factors.^22^ However, the pathophysiology of deafness‐associated mt‐tRNA mutations was far less understood.

To understand the molecular mechanism underlying mitochondrial deafness, recently, we carried out a mutational analysis for deafness‐related m.1555A>G and m.1494C>T mutations by using a novel multiplex allele‐specific PCR (MAS‐PCR) in 500 patients with NSHL and 300 controls from five hospitals from Zhejiang Province.^23^, ^24^ We first designed four primers that specifically binding to human 12S rRNA gene, after PCR amplification and electrophoresis, patients carrying the m.1555A>G mutation resulted in two specific bands: 736‐bp and 226‐bp, while subjects with the m.1494C>T mutation created two bands: 736‐bp and 488‐bp, whereas patients without these primary mutations can amplify only one band: 736‐bp, which was consistent with PCR‐Sanger sequencing.^25^ During that process, we ascertained a Chinese pedigree with NSHL. Screening for the entire mitochondrial genome suggested the coexistence of tRNA^Ala^ 5601C>T and tRNA^Leu(CUN)^ 12311T>C mutations. To further explore the contributions of mtDNA mutations to deafness expression, we analyzed the ATP, MMP, and ROS levels from the patients harboring these mtDNA mutations. We also performed the mutational analysis of *GJB2* and *SLC26A4* genes in matrilineal relatives of this pedigree.

## 2 MATERIALS AND METHODS

### 2.1 Family information and clinical examinations

**FIGURE 1:**
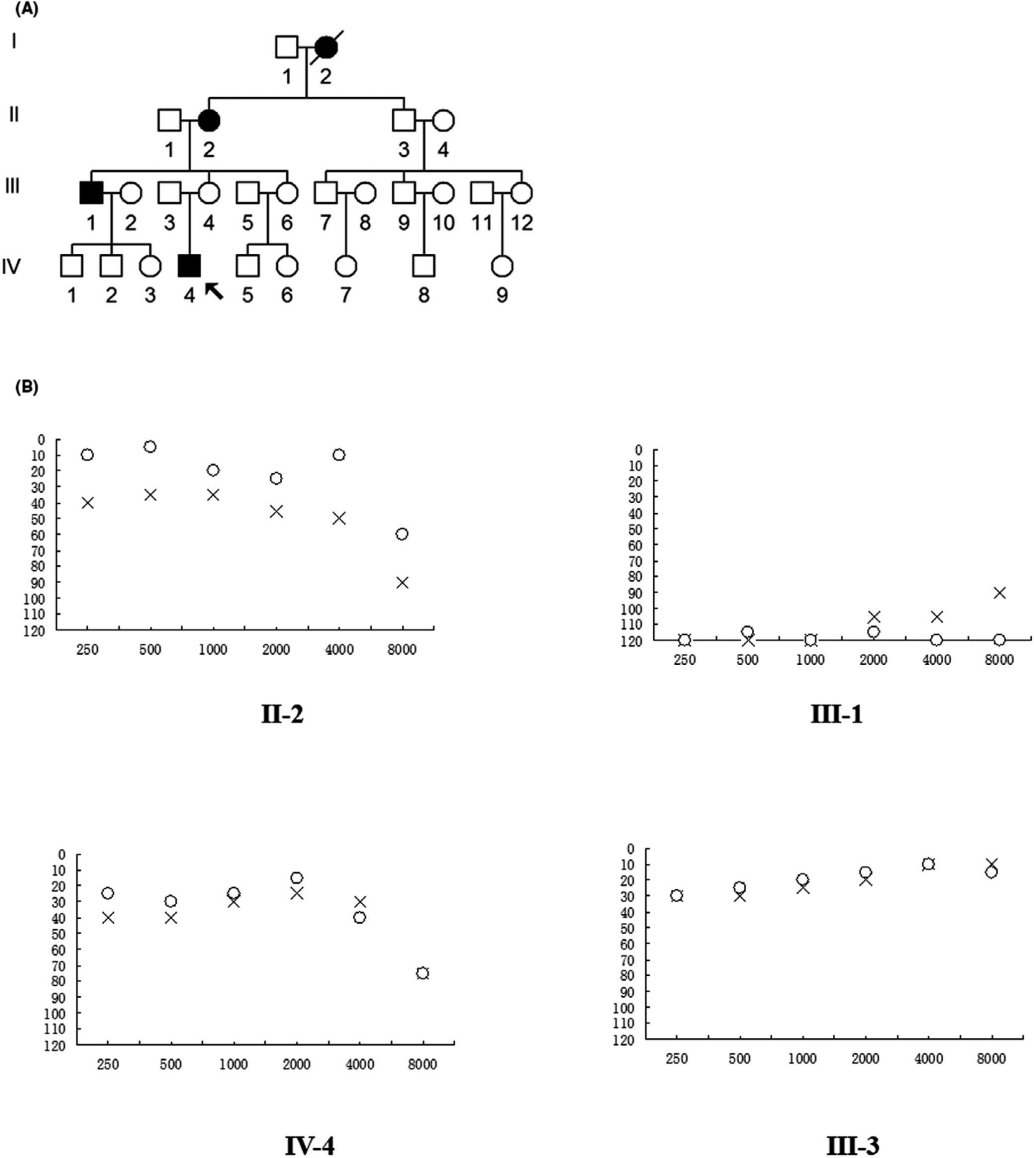
(A) Pedigree of a NSHL family with m.5601C>T and m.12311T>C mutations, arrow indicates the proband, hearing‐impaired individuals are indicated by filled symbols. (B) Air conduction audiogram of four members of this Chinese family. X, left ear; O, right ear

We ascertained a Han Chinese family in the Department of Otolaryngology, Quzhou People's Hospital (Figure 1A). Among nine matrilineal members, four of them were deaf patients (I‐2, II‐2, III‐1, and IV‐4). The blood samples, detailed demographics, and medical history such as the use of aminoglycosides antibiotics (AmAn) were obtained from these subjects of this family, this study was approved by the Ethical Committee of Quzhou People's Hospital, and the written informed consent was provided by each family member. Moreover, 300 healthy subjects including 169 males and 131 females were recruited as controls.

In addition, the pure tone audiometric (PTA) was carried out according to a previous investigation.^26^ We further measured the values of PTA based on the average of the hearing level at 0.25, 0.5, 1.0, 2.0, 4.0, and 8.0 kHz for each ear. The degrees of hearing loss were categorized as five grades: PTA<26 decibels (dB): normal hearing; PTA ranged between 26 and 40 dB: mild hearing loss; PTA ranged between 41 and 70 dB: moderate hearing loss; PTA ranged between 71 and 90 dB: severe hearing loss; and PTA>90 dB: profound hearing loss.

### 2.2 mtDNA genome sequencing

To explore the contributions of mtDNA mutations to deafness expression, the total genomic DNA from the family members (II‐2, III‐1, and IV‐4), together with 300 controls were isolated by the DNA extraction kit (Qiagen, Hilden, Germany). The complete mtDNA genes were amplified by 24 primers.^27^ The amplified fragments were sequenced and analyzed by comparing with the reversed Cambridge Reference Sequences (rCRS, GenBank accessible No: NC_012920.1).^28^ The DNAstar software (version 3.0) was used to analyze data.

### 2.3 Analysis of conservation of mtDNA mutations

To detect the deafness‐related pathogenic mtDNA mutations, phylogenetic analysis was performed. In brief, 13 species’ mtDNA sequences were used for this alignment. The conservation index (CI) was measured by using Clustal W software (http://www.clustal.org/).^29^ If the CI≥75%, we regarded it as having functional potential.^30^

### 2.4 Classification of mtDNA haplogroup

The mtDNA haplogroup was classified according to the phylotree (http://www.phylotree.org/) and the report by Kong et al.^31^

### 2.5 PMNs isolation

The PMNs from three subjects with hearing loss (II‐2, III‐1, and IV‐4), as well as three healthy individuals (III‐3, III‐5, and III‐8) from this family, were isolated using the method as described in our previous study.^32^

### 2.6 ATP analysis

The Cell Titer‐Glo^®^ Luminescent Cell Viability Assay kit (Promega, Madison, USA) was used to determine the ATP production in mutant cell lines carrying tRNA mutations and the controls, using the protocol provided by the manufacturer.^33^

### 2.7 MMP measurement

Decreased in MMP was the early biological event for program cell death.^34^ For MMP measurement, the mutant and control cells lines were first treated with the fluorescent probe, after 30‐min reaction; the fluorescence plate reader was used to determine the MMP.

### 2.8 ROS analysis

Since mitochondria generated ATP and released ROS as a toxic byproduct. To analyze ROS level, cells were firstly treated with the fluorescent probe 2,7‐dichlorodihydrofluorescein (DCFH) for 30 min, then the fluorescence plate reader was employed to qualify ROS production. ^35^

### 2.9 Screening for *GJB2* mutations

Mutations in *GJB2* were associated with hearing impairment.^36^ To assess whether *GJB2* contributed to the phenotypic expression of hearing loss, a mutational screening of *GJB2* was performed. The primers for PCR amplification of *GJB2* were forward, 5’‐TATGACACTCCCCAGCACAG‐3’, and reverse, 5’‐GGGGCAATGCTTAAACTGGC‐3’.^37^ After PCR, the products were sequenced, and the data were handled by DNAstar software (version 3.0) to detect the mutations.

### 2.10 Genotyping analysis of *SLC26A4* gene

To assess whether *SLC26A4* played an active role in deafness expression, a mutational screening for *SLC26A4* was performed in the matrilineal relatives in this pedigree (II‐2, III‐1, and IV‐4). The five primer sequences for *SLC26A4* were as follows: forward, 5’‐CGTGTAGCAGCAGGAAGTAT‐3’, and reverse, 5’‐TTAAATAAAAAAGACTGACT‐3’; forward, 5’‐TGGGGAAAAAGG ATGGTGGT‐3’, and reverse, 5’‐CCAACCCCTTCTTTAGCTGA‐3’; forward, 5’‐GCAGGATAGCTCAAGGAATT‐3’, and reverse, 5’‐TCATCA GGGAAAGGAAATAA‐3’; forward, 5’‐TCTCCTTGATGTCTTGCT TA‐3’, and reverse, 5’‐CCCATGTATTTGCCCTGTTG‐3’; and forward, 5’‐CTGGGCAATAGAATGAGACT‐3’, and reverse, 5’‐ATCTGTAGAAAGGTTGAATA‐3’.^38^ The sequence data were compared with the wide‐type version of *SLC26A4* (GenBank accessible No: NM_000441.1) to detect mutations.

### 2.11 Computer analysis

The Student's t‐test was used to determine the statistical importance, *p* < 0.05 was regarded to be statistically significant.

## 3 RESULTS

### 3.1 Clinical characterization of one pedigree with NSHL

We enrolled a maternally inherited family with NSHL, as shown in Figure 1A, the proband (IV‐4), aged 24, suffered from NSHL three years ago and came to Quzhou People's Hospital for treatment of deafness. As indicated in Figure 1B, the audiological examinations revealed that he developed the moderate NSHL (40 dB at left ear and 35 dB at right ear).

As shown in Figure 1A, four of nine matrilineal members in this family expressed NSHL as sole clinical phenotype, without any other diseases including cardiovascular, muscular, neurological, or endocrine diseases. As shown in Table 1, further genetic counseling suggested that the proband's uncle (III‐1) and grandmother (II‐2) also developed NSHL. In particular, the subjects (III‐1 and II‐2) had profound NSHL (110 dB at the left ear and 108 dB at the right ear; 103 dB at the left ear and 99 dB at the right ear, respectively). Further medical history revealed that subject (I‐2) was also a deaf patient who died three years ago. However, no members in this pedigree had any history of using AmAn, and other members in this pedigree had normal hearing (Figure 1B).

**TABLE 1:** Summary of clinical and molecular data for several members in this pedigree

Subject	Gender	Age at test (Year)	Age at onset (Year)	Ototoxic drug	PTA (dB) Left ear	PTA (dB) Right ear	Audiometric configuration	Level of hearing loss	Presence of mt‐tRNA mutations
II−2	Female	75	60	No	103	99	Slope	Profound	tRNA^Ala^ 5601C>T and tRNA^Leu(CUN)^ 12311T>C
III−1	Male	50	48	No	110	108	Slope	Profound	tRNA^Ala^ 5601C>T and tRNA^Leu(CUN)^ 12311T>C
IV−4	Male	24	21	No	40	35	Flat	Mild	tRNA^Ala^ 5601C>T and tRNA^Leu(CUN)^ 12311T>C
III−3	Male	55	/	No	21	19	Flat	Normal	None

Abreviations: dB, decibels; PTA, pure tone audiometry.

### 3.2 Mutational screening for mtDNA

**FIGURE 2:**
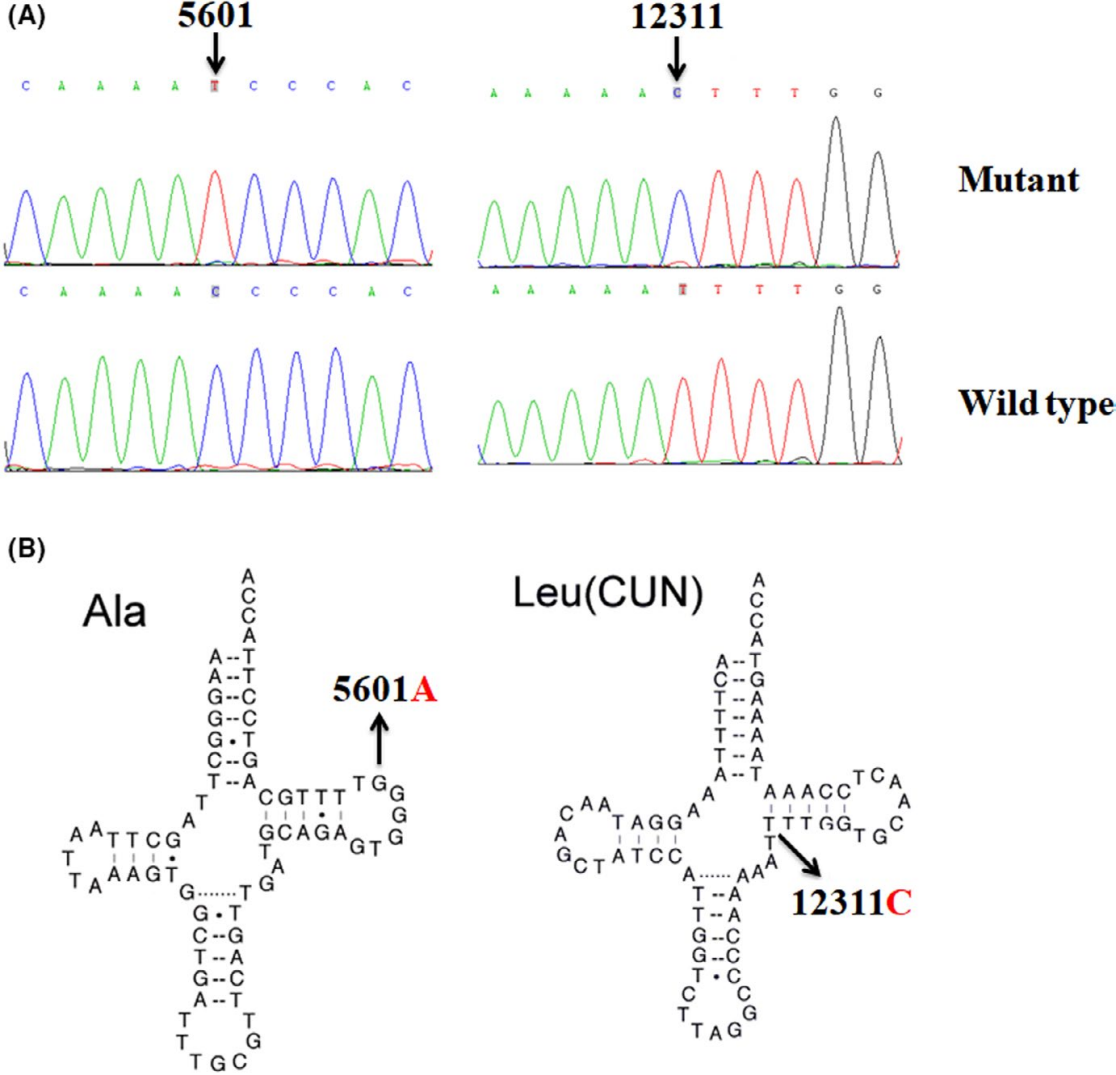
(A) Identification of m.5601C>T and m.12311T>C mutations by using PCR‐Sanger sequencing. (B) The locations of m.5601C>T in tRNA^Ala^ gene and m.12311T>C mutation in tRNA^Leu(CUN)^ gene

**FIGURE 3:**
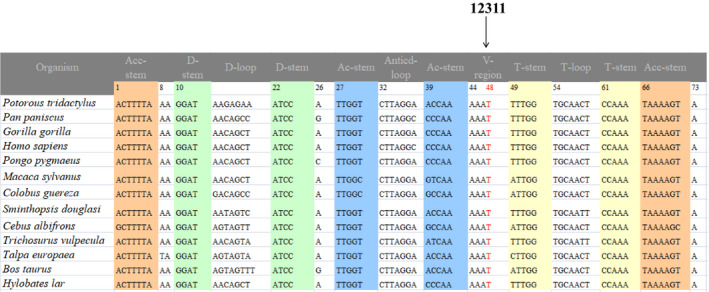
Alignment of tRNA^Leu(CUN)^ gene from different species, arrow indicates the location of m.12311T>C mutation

The entire mitochondrial genomes from the matrilineal relatives (II‐2, III‐1, and IV‐4) and 300 controls were PCR amplified and sequenced. Compared with the rCRS,^28^ members of this pedigree exhibited 36 variants, which belonged to mtDNA haplogroup G2b2.^31^ As summarized in Table 2, ten variants were identified in D‐loop, three variants were found in 12S rRNA, two variants occurred at 16S rRNA, two mutations in tRNA (tRNA^Ala^ 5601C>T and tRNA^Leu(CUN)^ 12311T>C) and the rest of the variations were mainly located at respiratory chain coding genes. Moreover, six missense variations were as follows: *ND1* 4048G>A (p. Asp248Asn), *A6* 8584G>A (p. Ala20Thr) and 8860A>G (p. Thr112Ala), *ND5* 13928G>C (p. Ser531Thr), *CytB* 14766C>T (p. Thr7Ile), and 15326A>G (p. Thr194Ala). These protein‐coding genes mutations, as well as tRNAs mutations, were evaluated by evolutionary conservation analysis including mouse,^39^ bovine,^40^ and *Xenopus laevis*.^41^ As shown in Figures 2A and 3, we found that only the m.5601C>T in tRNA^Ala^ and m.12311T>C in tRNA^Leu(CUN)^ showed high level of conservation (CI = 100% for all).

**TABLE 2:** mtDNA variants in this family with hearing loss

Gene	Nucleotide position	Replacement	Amino acid change	Conservation (H/B/M/X)^a^	rCRS^b^	GenBank frequency^c^	Classification
D‐loop	73	A to G			A	0.76	Benign
	150	C to T			C	0.166	Benign
	204	T to C			T	0.066	Benign
	215	A to G			A	0.0082	Benign
	263	A to G			A	0.948	Benign
	310	T to TC			T	0.00	Benign
	16093	T to C			T	0.0531	Benign
	16183	A to C			A	0.0047	Benign
	16223	C to T			C	0.181	Benign
	16519	T to C			T	0.631	Benign
12S rRNA	709	G to A		G/A/A/–	G	0.146	Benign
	750	A to G		A/A/A/G	A	0.983	Benign
	1438	A to G		A/A/A/G	A	0.968	Benign
16S rRNA	2706	A to G		A/G/A/A	A	0.79	Benign
	3107	del C		C/T/T/T	C	0.00004	Benign
*ND1*	3759	A to G			A	0.00032	Benign
	3970	C to T			C	0.037	Benign
	4048	G to A	Asp to Asn	D/N/Y/F	G	0.0058	Benign
*ND2*	4769	A to G		M/M/M/I	A	0.977	Benign
	4883	C to T			C	0.0109	Benign
tRNA^Ala^	5601	C to T		C/C/C/C	C	0.0138	Pathogenic
*CO1*	7028	C to T			C	0.809	Benign
*A6*	8584	G to A	Ala to Thr	A/V/V/I	G	0.0212	Benign
	8860	A to G	Thr to Ala	T/A/A/T	A	0.987	Benign
*ND3*	10310	G to A			G	0.00014	Benign
*ND4*	11719	G to A			G	0.71	Benign
	11914	G to A			G	0.108	Benign
tRNA^Leu(CUN)^	12311	T to C		T/T/T/T	T	0.0015	Pathogenic
*ND5*	12705	C to T			C	0.418	Benign
	12882	C to T			C	0.00409	Benign
	13928	G to C	Ser to Thr	S/T/S/T	G	0.0269	Benign
*ND6*	14311	T to C			T	0.00113	Benign
*CytB*	14766	C to T	Thr to Ile	T/S/T/S	C	0.77	Benign
	14783	T to C			T	0.0535	Benign
	15301	G to A			G	0.287	Benign
	15326	A to G	Thr to Ala	T/M/I/I	A	0.987	Benign

^a^ Conservation of amino acid for polypeptides or nucleotide for rRNAs, in human (H), mouse (M), bovine (B), and *Xenopus laevis* (X).

^b^ rCRS: reversed Cambridge Reference Sequence.

^c^ Please refer to Mitomap (https://www.mitomap.org/MITOMAP) database.

To screen the potential pathogenic mt‐tRNA mutations, the following criteria were used: (1) the allele frequency was <1% in the controls; (2) had a high level of evolutionary conservation^30^; and (3) may impair the mitochondrial functions.

As shown in Table 3 and Figure 2B, m.5601C>T mutation was present in homoplasmic form and occurred at TψC loop of tRNA^Ala^ (position 59), while the m.12311T>C mutation occurred at extremely conserved nucleotide in the connection between variable region and TψC loop of tRNA^Leu(CUN)^ (Figure 2B).^42^ Further analysis indicated that these tRNA mutations were found in all matrilineal members, but absent in other individuals of this pedigree and in 300 controls.

**TABLE 3:** Molecular features of mt‐tRNA^Ala^ 5601C>T and tRNA^Leu(CUN)^ 12311T>C mutations

tRNA species	Nucleotide changes	Number of nucleotides in tRNA	Location in tRNA	CI (%)	Disease association
tRNA^Ala^	5601C>T	59	TψC loop	100	LHON; hypertension; deafness
tRNA^Leu(CUN)^	12311T>C	48	Variable region	100	CPEO

Abbreviations: CI, conservation index; CPEO, chronic progressive external ophthalmoplegia; LHON, Leber's Hereditary Optic Neuropathy.

### 3.3 m.5601C>T and m.12311T>C affected ATP synthesis

**FIGURE 4:**
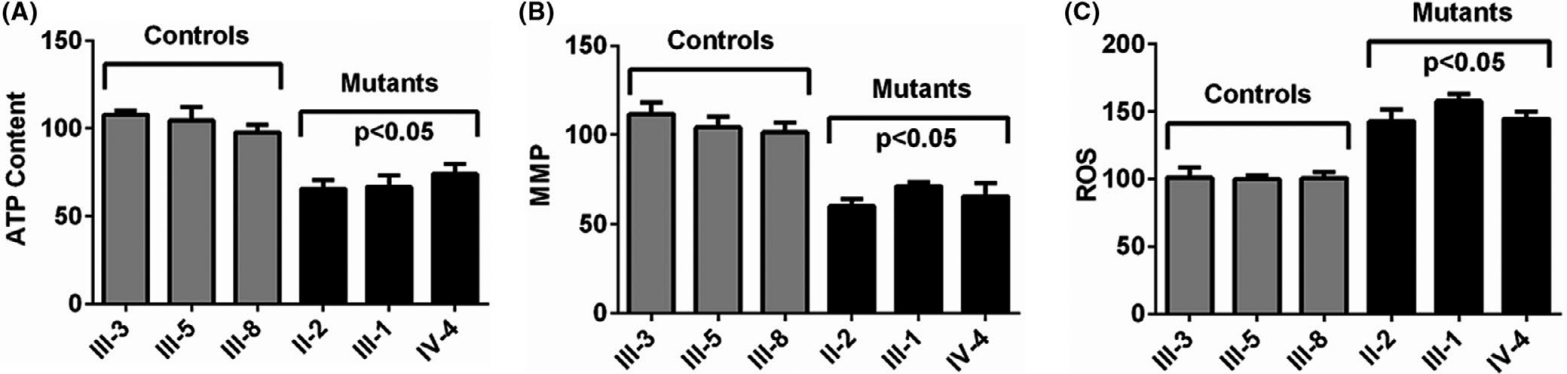
Mitochondrial functional analysis: (A) analysis of ATP level in three subjects with hearing loss and three controls; (B) MMP analysis; (C) determining the ROS level

To see whether m.5601C>T and m.12311T>C mutations affected mitochondrial functions, the PMNs of three patients (II‐2, III‐1, and IV‐4) with hearing loss and three controls (III‐3, III‐5, and III‐8) without these mutations were isolated and further used to analyze the mitochondrial functions. Almost ~30% drop in ATP synthesis was found in the mutant cells as compared to the controls (Figure 4A, *p* < 0.05).

### 3.4 MMP decreased significantly

The cells containing m.5601C>T and m.12311T>C mutations had a much lower level of MMP when compared to controls without these mutations (Figure 4B, *p* < 0.05).

### 3.5 Increase in ROS production

As shown in Figure 4C, patients with the m.5601C>T and m.12311T>C mutations exhibited much higher level of ROS production than the controls (*p *< 0.05).

### 3.6 Mutational analysis of *GJB2* gene

To see whether *GJB2* mutations played active roles in clinical expression of NSHL, we screened the mutations in the coding region of *GJB2*. However, we did not find any functional mutations in this gene.

### 3.7 Mutational analysis of *SLC26A4* gene

To explore the contributions of *SLC26A4* gene mutations to hearing impairment, the exons of *SLC26A4* were PCR amplified and sequenced. However, we failed to detect any variants in this gene.

## 4 DISCUSSION

In the present study, we identified two possible pathogenic mtDNA mutations: m.5601C>T in tRNA^Ala^ and m.12311T>C in tRNA^Leu(CUN)^ that caused hearing loss. The m.5601C>T and m.12311T>C were only found in matrilineal relatives but not detected in any other subjects of this family, as well as in 300 controls. In fact, m.5601C>T mutation occurred at position 59, which was extremely conserved from bacteria to human mitochondrion. In fact, mutation at that position was involved in the biochemical and molecular interactions between the TψC loop and D‐arm.^43^ Moreover, m.5601C>T mutation created a new base pairing (55T‐59C). RNAfold webserver showed that m.5601C>T altered the structure of tRNA^Ala^ ^44^; therefore, the mutant tRNA^Ala^ carrying this mutation may be more instable when compared to the wild‐type version of tRNA^Ala^. Previous studies suggested that the m.5601C>T mutation influenced the Leber's Hereditary Optic Neuropathy (LHON)‐related primary mutation in Han Chinese family^45^ and enhanced the expressivity of hypertension‐related tRNA^Met^ 4435A>G mutation.^44^

Moreover, T‐to‐C substitution at 12311 was first reported in patients with chronic progressive external ophthalmoplegia (CPEO).^46^ This mutation, however, resided at position, which was the connector between variable region and TψC stem in tRNA^Leu(CUN)^ (Figure 2B); importantly, the m.12311T>C caused the disruption of very conserved Watson–Crick base pairing (48T‐64A). It was implicated that the molecular interactions between nucleotides 15 and 48 played a significant role in the tRNA 3D structure; nucleotides alternations in either of these positions will affect tRNA functions.^47^ Interestingly, the m.12311T>C mutation increased the aminoacylation ability of tRNA^Leu(CUN)^ and affected its structure and function according to a recent study.^48^

In addition, mutations in *GJB2* and *SLC26A4* genes were associated with NSHL.^49^, ^50^ To understand the contributions of nuclear gene mutations to hearing loss, we screened the mutations in *GJB2* and *SLC26A4* genes, but no variants were identified.

It was well‐known that mtDNA genetic background (haplogroup) may modulate the clinical expression of NSHL. For instance, in pedigrees under haplogroups D4a, M22, and H2 harboring NSHL‐associated m.1555A>G or m.1494C>T mutations had much higher penetrance than those only carrying deafness‐associated primary mtDNA mutations.^19^ Moreover, mtDNA haplogroup B was found to enhance the risk of Eastern Asian pedigrees carrying m.1555A>G mutation,^51^ while the mtDNA haplogroup‐specific mutations tRNA^Thr^ 15927G>A of haplogroup B5b, CO1/tRNA^Ser(UCN)^ 7444G>A of haplogroup B4, tRNA^Cys^ 5802T>C, tRNA^Arg^ 10454T>C of haplogroup D4, and tRNA^Glu^ 14693A>G of haplogroup Y2 may increase the expressivity of NSHL in Chinese pedigrees with deafness‐associated 12S rRNA mutations.^14^ Sequence characterization of the mtDNA genes of family members indicated the presence of 36 variations allowed it to be assigned to haplogroup G2b2.^31^ To explore the influence of mtDNA haplogroups on deafness expression, a total of 23 pedigrees of NSHL were summarized in Table 4, which were associated with mt‐tRNA mutations. We found that the following mt‐tRNA mutations such as tRNA^Ile^ 4317A>G, tRNA^Thr^ 15924A>G, 15926C>T, 15927G>A, 15942T>C and 15940delT, tRNA^Leu(CUN)^ 12235T>C, tRNA^Gly^ 10019C>T and 10055A>G, tRNA^Leu(UUR)^ 3236A>G, tRNA^His^ 12192G>A and 12201T>C, tRNA^Phe^ 593T>C, tRNA^Ala^ 5587T>C and 5655T>C, tRNA^Asp^ 7551T>C, CO1/tRNA^Ser(UCN)^ 7444G>A, tRNA^Ser(UCN)^ 7445A>G, 7492C>T, 7471delG, 7496G>A, 7505T>C and 7511T>C, and tRNA^Lys^ 8339A>G and 8344A>G mutations may directly lead to hearing loss.^16^, ^19^, ^52^, ^53^, ^54^, ^55^, ^56^, ^57^, ^58^, ^59^, ^60^, ^61^, ^62^, ^63^

**TABLE 4:** Summary of clinical and molecular data for 23 pedigrees with nonsyndromic hearing loss carrying the primary mt‐tRNA mutations

Pedigree number	Country	Number of matrilineal relatives	Number of affected individuals	Penetrance of hearing impairment (%)	mt‐tRNA mutations	mtDNA haplogroup	References
1	China	8	3	37.5	tRNA^Ala^ 5601C>T and tRNA^Leu(CUN)^ 12311T>C	G2b2	This study
2	China	3	3	100	tRNA^Ile^ 4317A>G and tRNA^Thr^ 15924A>G	D4e1a	^16^
3	China	3	2	66.7	tRNA^Leu(CUN)^ 12235T>C and tRNA^Thr^ 15940 delT	Z4a	^16^
4	China	7	4	57.1	tRNA^Thr^ 15926C>T	B4c1b2a1	^16^
5	China	8	3	37.5	tRNA^Gly^ 10019C>T	D4j15	^16^
6	China	9	3	33.3	tRNA^Gly^ 10055A>G	M7b1a1	^16^
7	China	8	3	37.5	tRNA^Lys^ 8296A>G and tRNA^Ala^ 5587T>C	F1e	^16^
8	China	14	4	28.6	tRNA^Leu(UUR)^ 3236A>G and tRNA^Thr^ 15927G>A	G3b2	^16^
9	China	10	5	50	tRNA^His^ 12192G>A and tRNA^Thr^ 15927G>A	B5b1b	^52^
10	China	9	5	55.5	tRNA^Phe^ 593T>C	G2a2a	^53^
11	China	32	16	50	tRNA^His^ 12201T>C	Z3	^54^
12	China	9	7	77.7	tRNA^Ser(UCN)^ 7505T>C and tRNA^Ala^ 5587T>C	F1	^55^
13	China	16	6	37.5	tRNA^Asp^ 7551T>C	A4	^56^
14	Greece	7	1	14.3	COI/tRNA^Ser(UCN)^ 7444G>A	B4	^57^
15	China	8	1	12.5	tRNA^Ser(UCN)^ 7492C>T	G2b	^58^
16	Poland	10	3	30	tRNA^Ser(UCN)^ 7511T>C	Unknown	^59^
17	China	12	8	66.7	tRNA^Ser(UCN)^ 7511T>C and tRNA^Ala^ 5655T>C	Unknown	^60^
18	China	13	3	23.1	tRNA^Ser(UCN)^ 7471delG and tRNA^Leu(CUN)^ 12280A>G	G2a	^18^
19	China	6	2	33.3	CO1/tRNA^Ser(UCN)^ 7444G>A and tRNA^Thr^ 15942T>C	N9a	^18^
20	China	14	3	21.4	tRNA^Ser(UCN)^ 7496G>A	F1	^18^
21	Poland	12	7	58.3	tRNA^Ser(UCN)^ 7445A>G	H6	^61^
22	China	8	3	37.5	tRNA^Lys^ 8339A>G	F1a	^62^
23	USA	37	16	43.2	tRNA^Lys^ 8344A>G	Unknown	^63^

The mtDNA encoded the core subunits of the multiple polypeptide OXPHOS complexes I, III, IV, and V. Admixture of two different sets of mtDNA mutations (heteroplasmic forms) for the same OXPHOS polypeptide could be deleterious because different ratios of mutant and wild‐type mtDNA substantially affected disease expression and severity.^64^ However, some pathogenic mutations were in homoplasmic forms, as in the case of tRNA^Thr^ 15927G>A mutation,^65^ but homoplasmic mtDNA mutation was insufficient to produce the clinical phenotype, and needed additional modified factors such as nuclear genes and environmental factors.^66^

To see whether m.5601C>T and m.12311T>C mutations influenced mitochondrial functions, the PMNs of three subjects (II‐2, III‐1, and IV‐4) with hearing loss, together with three healthy subjects (III‐3, III‐5, and III‐8), were isolated. We found that, compared with the controls, ~30% reduction in ATP synthesis in PMNs with both m.5601C>T and m.12311T>C mutations was much lower than the diabetes‐associated tRNA^Leu(UUR)^ 3243A>G mutation.^67^ Furthermore, patients with m.5601C>T and m.12311T>C mutations exhibited much lower MMP than controls (~42% reduction), which was similar to tRNA^Lys^ 8344A>G mutation.^68^ These biological events may enhance the ROS level in PMNs with both m.5601C>T and m.12311T>C mutations; as a result, the overloaded ROS would lead to oxidative stress, damage mitochondrial and nucleic acids, and cause mitochondrial dysfunction.^69^ Thus, the m.5601C>T and m.12311T>C mutations may affect the cochlear cell death and apoptosis,^70^ thereby leading to the phenotypic expression of NSHL in this pedigree.

In conclusion, our study indicated that m.5601C>T and m.12311T>C mutations may be associated with NSHL in this family. Mt‐tRNA^Ala^ 5601C>T and tRNA^Leu(CUN)^ 12311T>C mutations should be regarded as pathogenic mutations for NSHL. Therefore, our study provided new information on clinical diagnosis, prevention, and treatment for mitochondrial deafness.

## CONFLICT OF INTEREST

None.

## Data Availability

All data generated or analyzed during this study are included in this article. Further enquiries can be directed to the corresponding author.
